# Supplementary material for: Structure and Undulations of Escin Adsorption Layer at Water Surface Studied by Molecular Dynamics
Source: Molecules. 2021 Nov 13;26(22):6856. doi: 10.3390/molecules26226856 (PMC8618613; doi:10.3390/molecules26226856)
Supplement: Supplementary file 1 [file molecules-26-06856-s001.zip › molecules-1439398-supplementary.pdf]

molecules

Supporting Information

Structure and Undulations of Escin Adsorption Layer at Water Surface Studied by Molecular Dynamics

Sonya Tsibranska [1], Anela Ivanova [2,*], Slavka Tcholakova[1] and Nikolai Denkov [1]

[1] Department of Chemical and Pharmaceutical Engineering, Faculty of Chemistry and Pharmacy, University of Sofia; st@lcpe.uni-sofia.bg (S.T.); sc@lcpe.uni-sofia.bg (S.T.); nd@lcpe.uni-sofia.bg (N.D.)
[2] Department of Physical Chemistry, Faculty of Chemistry and Pharmacy, University of Sofia
* Correspondence: aivanova@chem.uni-sofia.bg; +359-898 953 598

Intermolecular orientation within the clusters

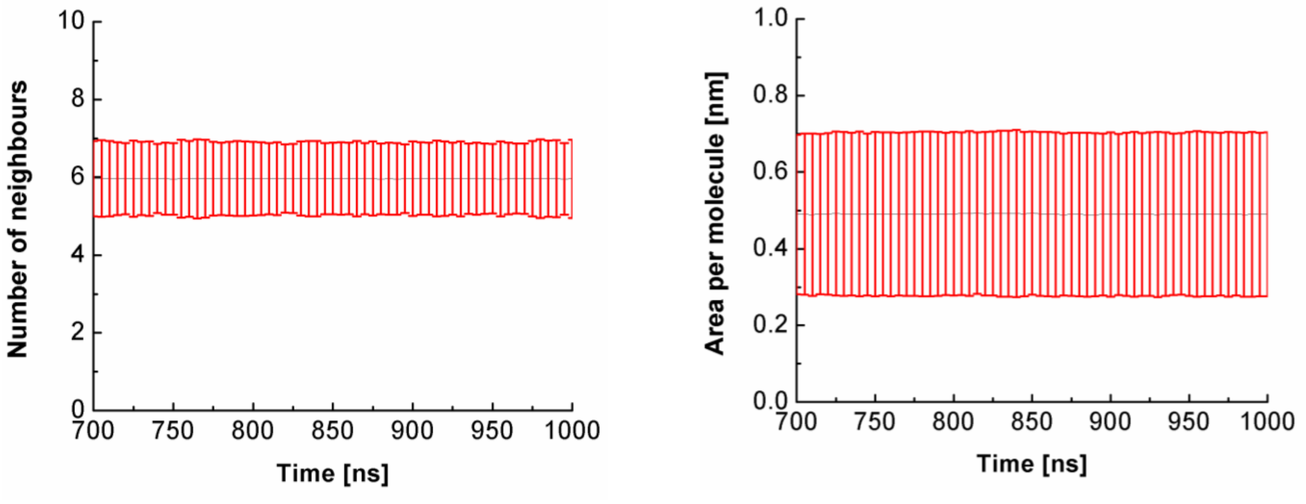

(A) (B)

Figure S1. Block averages (with standard deviations) of the number of nearest neighbors (**A**) and the area per molecule (**B**) of the escin surfactants during the last 300 ns of the simulation obtained from Voronoi analysis [89,90].

Model with 49 molecules(0.70 nm^2)

- Number of neighbors: 5.6 ± 1.5
- Area per molecule: 0.67 ± 0.17 nm^2

Model with 49 molecules(0.49 nm^2)

- Number of neighbors: 5.8 ± 0.8
- Area per molecule: 0.46 ± 0.11 nm^2
